# Yttrium (III) Recovery with D2EHPA in Pseudo-Emulsion Hollow Fiber Strip Dispersion System

**DOI:** 10.1038/s41598-018-25771-4

**Published:** 2018-05-16

**Authors:** Teerapon Pirom, Amornchai Arponwichanop, Ura Pancharoen, Tetsu Yonezawa, Soorathep Kheawhom

**Affiliations:** 1 Computational Process Engineering Research Laboratory, Department of Chemical Engineering, Faculty of Engineering, Chulalongkorn University, Bangkok, 10330 Thailand; 2 Division of Materials Science and Engineering, Faculty of Engineering, Hokkaido University, Sapporo, 060-8628 Japan

## Abstract

Yttrium (Y) is an essential lanthanide rare earth element and can be effectively extracted and purified using a hollow fiber supported liquid membrane (HFSLM) system. However, the stability of HFSLM system is a significant challenge. Pseudoemulsion-hollow fiber strip dispersion (PEHFSD) system, providing excellent stability, is attracting research attention. In this work, the recovery of Y(III) by PEHFSD system using di(2-ethylhexyl)phosphoric acid (D2EHPA) as a carrier was investigated. The effects of several operating parameters, including the initial concentration of Y(III) in the feed phase, the flow rate of feed, the stirring speed and the volumetric ratio of feed to strip on Y(III) separation were studied. The Y(III) transport was analyzed on the concentration ratio of Y(III) ions, percent extraction, percent stripping and overall mass transfer coefficient (*K*_*p*_). The PEHFSD system outperformed HFSLM system regarding separation performance and stability. *K*_*p*_ of HFSLM system decreased after the second run, but *K*_*p*_ of PEHFSD system remained constant even at the fifth run. The dispersed droplets in the strip dispersion phase in the PEHFSD system enhanced separation performance and stability of the membrane module.

## Introduction

Yttrium (Y) is a crucial lanthanide rare-earth element, widely used in various devices such as LCDs, electrodes, lasers, and superconductors. Besides, it is used in the synthesis of garnet and as an essential ingredient in several cancer medicines^1–3^. Also, Yttrium-90 is a pure beta-particle emitting radionuclide which is well suited to the applications in targeted therapy^4^.

In nature, yttrium is not found as a free element. However, it is always found in rare-earth minerals and uranium ores^5^. The concentration of yttrium in soil is observed between 10 and 150 ppm whilst its concentration is about 9 ppt in sea water^6^.

Yttrium from the mixed oxide ores can be purified by various techniques such as ion exchange, liquid-liquid extraction, and adsorption. Among these, liquid membrane techniques are promising due to their high cost-effectiveness, low energy and extractant consumption, and simplicity^7^. Also, these techniques benefit from non-equilibrium mass transfer and up-hill effect, where solute can transport across the membrane against their concentration gradient. Besides, the techniques can perform extraction and stripping simultaneously in the same stage.

Different types of liquid membrane systems such as emulsion liquid membrane (ELM)^8^, bulk liquid membrane (BLM)^9^, hollow fiber supported liquid membrane (HFSLM)^10^, flowing liquid membrane (FLM)^11^, electrostatic pseudo-liquid membrane (EPLM)^12^ and supported emulsion liquid membrane (SELM)^13^ have been proposed. However, the poor stability and difficult operations have constrained their large-scale industrial applications. In long-term, the liquid membrane can leak from the supporter. Consequently, the system performance is significantly decreased with time^14^.

In order to handle these issues, different configurations of HFSLM with strip/organic dispersion technique have been proposed. These configurations include hollow fiber renewal liquid membrane (HFRLM)^15^ and pseudo-emulsion hollow fiber strip dispersion (PEHFSD)^16^ system.

In PEHFSD system, a pseudo-emulsion of organic and strip phase flows through the shell-side whilst the feed phase runs in the tube-side. In the recent years, PEHFSD system is attracting much scientific interest as this technique presents coupled advantages of ELM and HFSLM. Also, PEHFSD system has been applied for the recovery of various metal ions i.e. Cu(II)^16,17^, Zn(II)^18,19^, Au(I)^20^, Co(II)^21^, Cr(III)^22,23^, Cr(VI)^24^ from aqueous solutions. However, to the best of our knowledge, the implementation of PEHFSD system to recover Y(III) from aqueous solution has not been previously reported.

This work aims at investigating the removal and recovery of Y(III) ions from nitrate solution using PEHFSD system. Di(2-ethylhexyl)phosphoric acid (D2EHPA) was employed as the carrier. The effects of various operating conditions including the feed flow rate, the initial concentration of Y(III) ion, the volumetric ratio of feed to stripping solution, the type of diluent and the stirring speed were examined. Besides, the performance and stability of PEHFSD system were also discussed and compared with that of a conventional HFSLM system.

## Experimental

### Materials

An analytical grade of Yttrium (III) oxide was procured from Sigma-Aldrich. D2EHPA (extractant) was purchased from Merck. The chemical structure of D2EHPA is presented in Fig. S1. Kerosene was procured from Shell. Analytical reagent grade of nitric acid (HNO3) and toluene (C7H8) were obtained from QRëC. All chemicals were used without further purification. Besides, all aqueous solutions were prepared using distilled water. The Y(III) solutions (1, 2 and 4 mM) were prepared by dissolving Yttrium (III) oxide in nitric acid.

### Apparatus

The experimental setup used for performing PEHFSD experiments is shown in Fig. S2. The system is comprised of two gear pumps with flow rate controllers, four pressure gauges, two rotameters, one overhead stirrer and one magnetic stirrer. Besides, the similar setup used for carrying out HFSLM experiments is shown in Fig. S3. For both systems, the hollow fiber modules (Liqui-Cel Extra-Flow module, Celgard), were employed to support the liquid membrane phase. The modules use micro-porous polypropylene fibers that are woven and wrapped around a focal tube feeder supplying the shell-side liquid. The woven fabric permits more uniform fiber spacing which promotes higher mass-transfer coefficient. The properties of the hollow fiber modules are presented in Table S1.

### Analytical instruments

The concentration of Y(III) in the feed and strip phases was measured by inductively coupled plasma optical emission spectrometer (ICP-OES, Perkin Elmer-Optima 5300 DV). The pH of the solutions was measured with pH meter (Mettler Toledo).

### Pseudo-Emulsion Hollow Fiber Strip Dispersion System

**Figure 1:**
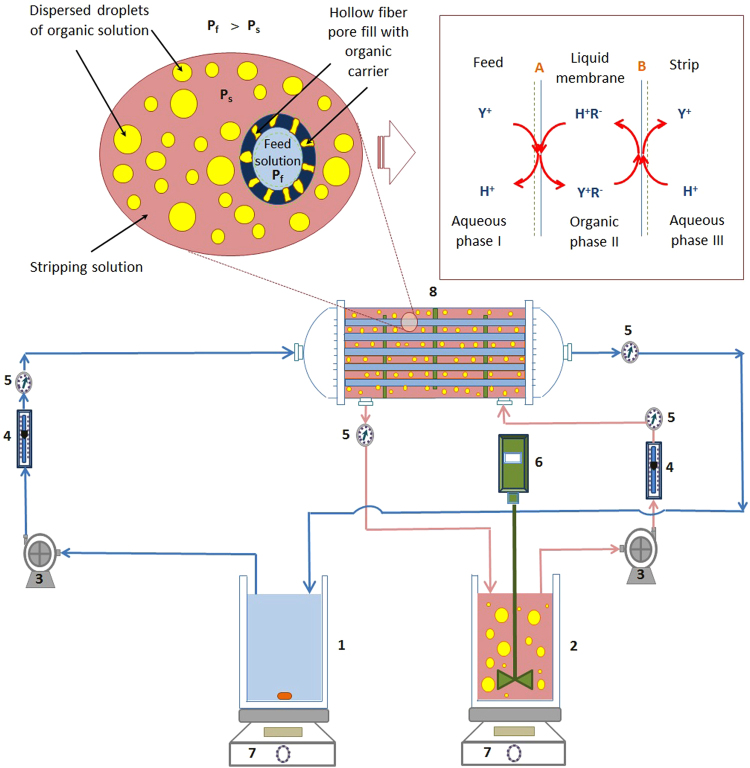
Schematic diagram of the PEHFSD system: (1) feed reservoir (2) stripping reservoir (3) gear pumps (4) rotameters (5) pressure gauges (6) overhead stirrer (7) magnetic stirrer and (8) hollow fiber module.

Figure 1 The experimental setup used for carrying out PEHFSD experiments is shown in Fig. 1. The aqueous strip solution was mixed with the organic phase containing the extractant. The use of a mixer helped disperse the aqueous strip solution in the organic phase. As the extractant is weak surface-active, small droplets of the aqueous strip solution dispersed in the continuous organic phase were formed. The pseudo-emulsion (the strip dispersion phase) was then fed into the shell-side of the hollow fiber module. Consequently, the organic phase readily wetted the micropores of the hydrophobic microporous support, and a stable liquid membrane in the micropores was formed. In comparison, the aqueous feed solution containing Y(III) flowed through the tube-side of the hollow fiber module. A small pressure difference between the aqueous feed solution side and the strip dispersion side was set at 30 kPa to prevent the leakage of the membrane through the micropores. The strip dispersion phase and feed phase were operated in the recycling mode in their respective reservoirs. As soon as the operation stopped, the strip phase and organic phase get separated. At various time intervals, samples of 0.5 mL of the aqueous phase were collected to determine the concentration of Y(III).

The volume of the pseudo-emulsion phase used in the experiment was 1,000 mL (500 mL of the organic phase and 500 mL of the strip solution). Also, the feed solution of 1,000 mL was employed. The flow rates of the feed phase and pseudo-emulsion phase were varied in the range of 100–300 mL/min.

The principle of PEHFSD system involves co-transportation and various equilibrium reactions which can be described by considering the upper-right inset picture of Fig. 1.

- Y(III) diffuses from the feed phase to the interface A.
- On the feed side interface of the PEHFSD, the extraction of Y(III) from the feed solution with D2EHPA (abbreviated as (HR2)) in kerosene can be expressed as (1).

1 $${{\rm{Y}}}_{{\rm{f}}}^{3+}+3{({\rm{H}}{\rm{R}})}_{\mathrm{2,}{\rm{org}}}\mathop{\mathop{\rightleftarrows }\limits^{{K}_{1}}}\limits_{{K}_{-1}}{\rm{Y}}{{\rm{R}}}_{3}{({\rm{H}}{\rm{R}})}_{\mathrm{3,}{\rm{org}}}+3{{\rm{H}}}_{{\rm{f}}}^{+}$$

where the subscriptions of f and org stand for the feed and organic phases, respectively. (HR)_2 indicates that the D2EHPA in kerosene mainly exists as a dimer. *K*_1_ and *K*_−1_ stand for forward and backward reaction rate constants at the interface between the feed and membrane phases.

- Y(III) complex YR3(HR)3 diffuses across the membrane A-B.
- At the stripping side interface of the PEHFSD, the YR3(HR)3 dissolves in the organic membrane solution, and the Y(III) ion are then stripped. The stripping reaction occurred on the other side of the membrane is shown in (2)^14,25^.

2 $$Y{R}_{3}{(HR)}_{\mathrm{3,}{\rm{org}}}+3{H}_{{\rm{s}}}^{+}\mathop{\mathop{\rightleftarrows }\limits^{{K}_{2}}}\limits_{{K}_{-2}}{Y}_{f}^{3+}+\mathrm{3(}HR{)}_{\mathrm{2,}{\rm{org}}}$$

where s represents the dispersion phase. *K*_2_ and *K*_*−*2_ stand for forward and backward reaction rate constants at the interface between the membrane and dispersion phases.

- Finally, D2EHPA returns from B to A. In this mechanism, the separation of Y(III) across PEHFSD is described by considering only the diffusion coefficient of Y(III) because the complex reaction between the Y(III) and D2EHPA at the interfaces is much faster than the diffusion in the feed and membrane phases^26–28^. The transport model of Y(III) operating in the recycling mode is considered by the overall mass transfer coefficient of Y(III) transport (*K*_p_)^29^. ${K}_{p}$ is derived based on (3–6).

- The component balance of [Y] in the membrane module (the feed side).
  3 $$\frac{d{[{\rm{Y}}]}_{{\rm{aq}}}^{{\rm{M}}}}{dt}=-\,{u}_{{\rm{aq}}}\frac{d{[{\rm{Y}}]}_{{\rm{aq}}}^{{\rm{M}}}}{dz}-{(\frac{A}{{V}_{{\rm{M}}}})}_{{\rm{in}}}{K}_{p}({[{\rm{Y}}]}_{{\rm{aq}}}^{{\rm{M}}}-{[{\rm{Y}}]}_{{\rm{str}}}^{{\rm{M}}})$$
- The component balance of [Y] in the feed tank.
  4 $$\frac{d{[{\rm{Y}}]}_{{\rm{aq}}}^{{\rm{T}}}}{dt}=\frac{{Q}_{{\rm{aq}}}}{{V}_{{\rm{aq}}}}([{\rm{Y}}{]}_{{\rm{aq}},z=L}^{{\rm{M}}}-{[{\rm{Y}}]}_{{\rm{aq}},z\mathrm{=0}}^{{\rm{M}}})$$
- The component balance of [Y] in the membrane module (the strip side).
  5 $$\frac{d{[{\rm{Y}}]}_{{\rm{str}}}^{{\rm{M}}}}{dt}=-\,{u}_{{\rm{str}}}\frac{d{[{\rm{Y}}]}_{{\rm{str}}}^{{\rm{M}}}}{dz}-{(\frac{A}{{V}_{{\rm{M}}}})}_{{\rm{out}}}{K}_{p}([{\rm{Y}}{]}_{{\rm{aq}}}^{{\rm{M}}}-{[{\rm{Y}}]}_{{\rm{str}}}^{{\rm{M}}})$$
- The component balance of [Y] in the strip tank.

6 $$\frac{d{[{\rm{Y}}]}_{{\rm{str}}}^{{\rm{T}}}}{dt}=\frac{{Q}_{{\rm{str}}}}{{V}_{{\rm{str}}}}([{\rm{Y}}{]}_{{\rm{str}},z=0}^{{\rm{M}}}-{[{\rm{Y}}]}_{{\rm{str}},z=L}^{{\rm{M}}})$$

In these equations, *L* is the length of the fiber. *Q* is the flow rate. *u* is the linear velocity. *V* is the volume of each phase. The superscripts *M* and *T* refer to the membrane module and tank, respectively. The subscripts aq and str refer to the feed and strip phases, respectively. Because of the assumption that the stripping reaction is instantaneous and occurs at the fiber interface, thus, the solution of the above equations is expressed as in (7).

7 $${V}_{{\rm{aq}}}{\rm{l}}{\rm{n}}\frac{{[{\rm{Y}}]}_{{\rm{aq}},t\mathrm{=0}}^{{\rm{T}}}}{{[{\rm{Y}}]}_{{\rm{aq}}}}={Q}_{{\rm{aq}}}[1-{e}^{-\frac{2{K}_{p}L}{{u}_{{\rm{aq}}}{r}_{i}}}]t=St$$

where *K*_*p*_ is the overall mass transfer coefficient. *r*_*i*_ is the inner radius of the hollow fiber, *S* is the slope of this linear relationship estimated from the experimental data. Therefore, *K*_*p*_ is explicitly expressed as in (8).

8 $${K}_{p}=-\,\frac{{u}_{{\rm{aq}}}{r}_{i}}{2L}\,\mathrm{ln}\,[1-\frac{S}{{Q}_{{\rm{aq}}}}]$$

## Results and Discussion

### Effect of the initial concentration of Y(III) in the feed

**Figure 2:**
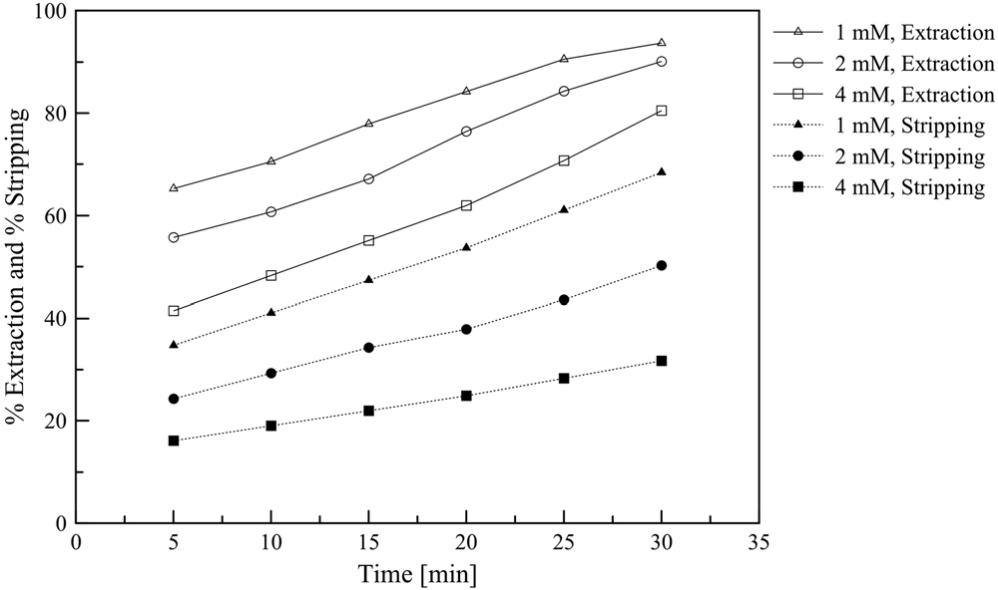
The effects of initial feed concentration on percent extraction and stripping versus time, feed agents of 0.5 M HNO_3_, stripping agents of 3.0 M HNO_3_, 0.1 M D2EHPA in kerosene, volumetric ratio feed to strip dispersion = 1, the flow rate of feed to stripping dispersion solution = 300:200 mL/min, stirring speed = 500 rpm and operating time = 30 min in circulating mode.

The transportation of Y(III) via the PEHFSD system at different initial concentrations of Y(III) in the feed (1, 2 and 4 mM) has been carefully studied. Figure 2 presents the variation of the percentages of extraction and stripping. The percent extraction and percent stripping were calculated using (9) and (10).

9 $$ \% {\rm{Extraction}}=\frac{{[{\rm{Y}}]}_{{\rm{aq}},t\mathrm{=0}}^{T}-{[{\rm{Y}}]}_{{\rm{aq}},t}^{T}}{{[{\rm{Y}}]}_{{\rm{aq}},t\mathrm{=0}}^{T}}\times 100$$

10 $$ \% {\rm{Stripping}}=\frac{{[{\rm{Y}}]}_{{\rm{str}},t}^{T}}{{[{\rm{Y}}]}_{{\rm{aq}},t\mathrm{=0}}^{T}}\times 100$$

In the initial 30 min of operation, both stripping and extraction percentages gradually increased with time. The initial concentration Y(III) of 1 mM exhibited the highest profile of percent extraction in the range of 62–93 whilst the profile of percent stripping was in the range of 34–63. The percentages of extraction and stripping decreased as the initial Y(III) concentration in the feed phase increases. In other words, the lower initial concentration of Y(III) showed the higher percentage of extraction and stripping. Other authors have reported a similar trend of permeation for the transport of Zn(II), Co(II), V(V) and U(IV) in the PEHFSD system^30–33^. This phenomenon could be explained that the organic phase within the membrane microspores became saturated with Y(III) complex by the initial concentration of Y(III) 1 mM in the feed phase. Moreover, the Y(III) complex diffuses slowly into the bulk of the organic solution resulting in a reduction in the mass transfer of Y(III) ion. Therefore, the percent extraction and stripping decreased at the initial concentration of Y(III) in the feed above 1 mM.

### Effect of the volumetric ratio of the feed to strip dispersion

**Figure 3:**
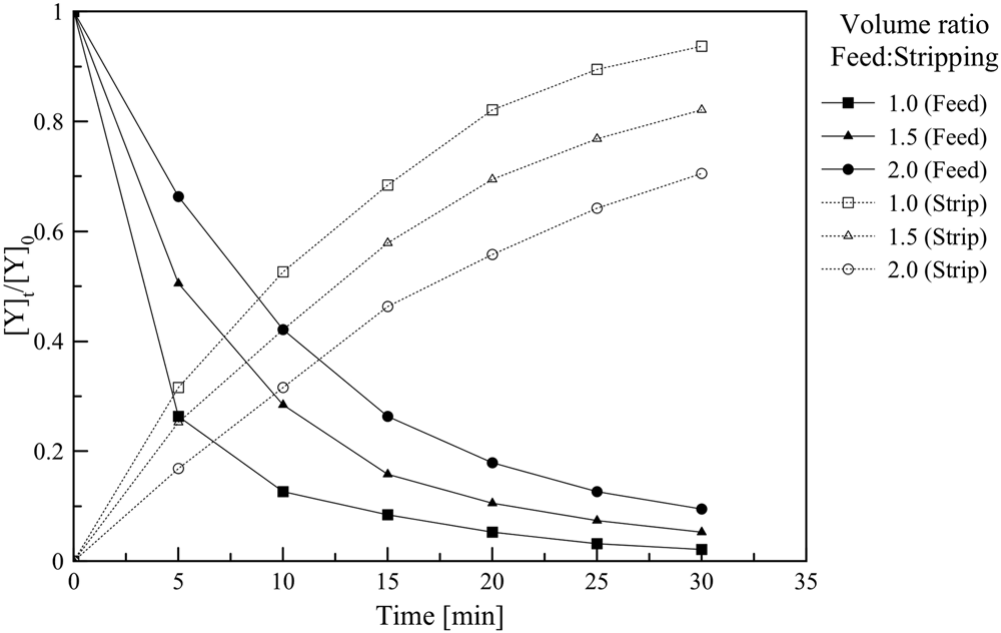
The effects of volumetric ratio of feed to strip dispersion on concentration ratio of Y(III) in the feed and strip phase versus time, Y(III) concentration in feed phase = 1 mM feed agents = 0.5 M HNO_3_, stripping agents = 3.0 M HNO_3_, D2EHPA concentration in kerosene = 0.1 M, the flow rate of feed: stripping dispersion solution = 300:200 mL/min, stirring speed = 500 rpm and operating time = 30 min in circulating mode.

Figure 3 presents the effects of the volumetric ratio of feed to strip dispersion on the concentration ratio of Y(III) in the feed ([Y]_aq,t_/[Y]_aq,t=0_) and in the strip ([Y]_str,t_/[Y]_str,t=0_). Here, the volumes of both strip and organic phases were set at 500 mL, and the volume of feed phase was varied 1.0, 1.5 and 2.0 L. The concentration ratio of Y(III) in the feed phase dropped with time whilst the concentration ratio in the strip increased with time. Moreover, in all cases, the rapid changes were observed at initial, and the profiles became flat at longer operation time. At high volumetric ratio, the concentration ratio in the strip was lower than that of low volumetric ratio due to the limit of the number of extractant molecules being available for the reaction with Y(III) ion. Also, the driving force of differential concentration is decreased as the volumetric ratio increases. This effect was also reported in previous studies on the permeation of Cu(II)^16^, Co(II)^31^ and Au(I)^34^ via PEHFSD system.

### Effect of organic diluent

**Figure 4:**
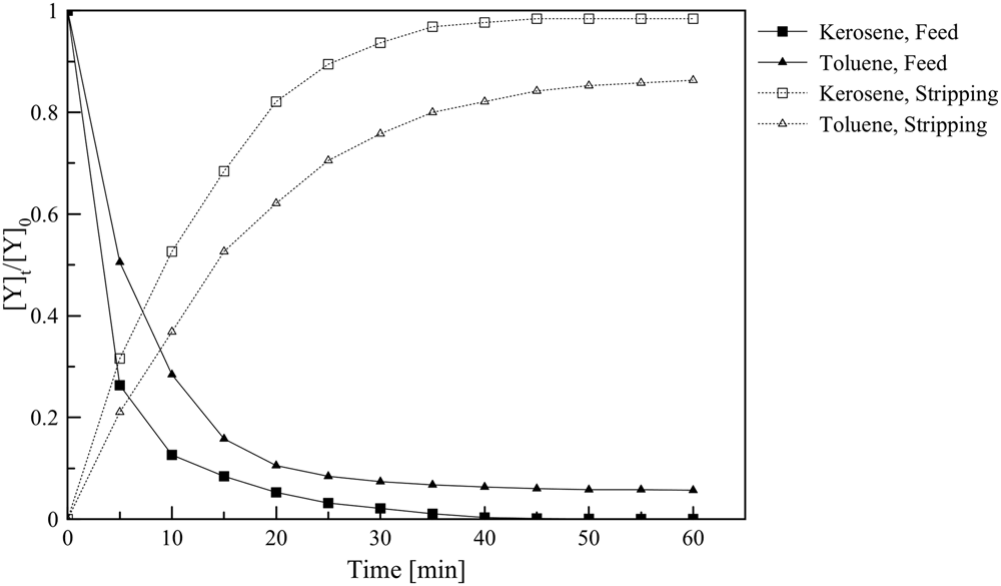
The effects of diluent on concentration ratio of Y(III) in feed and strip phase versus time, Y(III) concentration in feed phase = 1 mM feed agents = 0.5 M HNO_3_, stripping agents = 3.0 M HNO_3_, D2EHPA concentration = 0.1 M, the flow rate of feed stripping dispersion solution = 300:200 mL/min, stirring speed = 500 rpm and operating time = 60 min in circulating mode.

The organic diluent has a significant function in liquid membrane systems because it is the primary constituent of the membrane phase. Thus it affects physical properties of the liquid membrane^35^. A higher viscosity of the diluent can increase the emulsion stability, but a high viscosity has adverse effects on the mass transport and diffusivity. In comparison, lower viscosity of the diluent benefits the overall capacity and decreases mass-transport resistance. Besides, the polarity of the diluent also affects the efficiency of an extractant-carrier system in a membrane. In this study, two diluents with different polarity indexes, including kerosene (1.8) and toluene (2.4), were examined. Kerosene has been commonly employed due to its low viscosity, availability and non-polar characteristic. Polarity index of the diluent contributes to the molecular attraction with Y(III) complex. As shown in Fig. 4, the relationship between the polarity of the diluents and the concentration ratio of Y(III) indicated that kerosene is a suitable organic diluent for the extraction of Y(III). The lower polarity index of diluent facilitates the formation and reformation of Y(III) complex. Besides, the smaller polarity index of diluent also inhibits the formation of Y(III) complex in extract reaction and the reformation of yttrium complex in strip reaction.

### Effect of stirring speed

**Figure 5:**
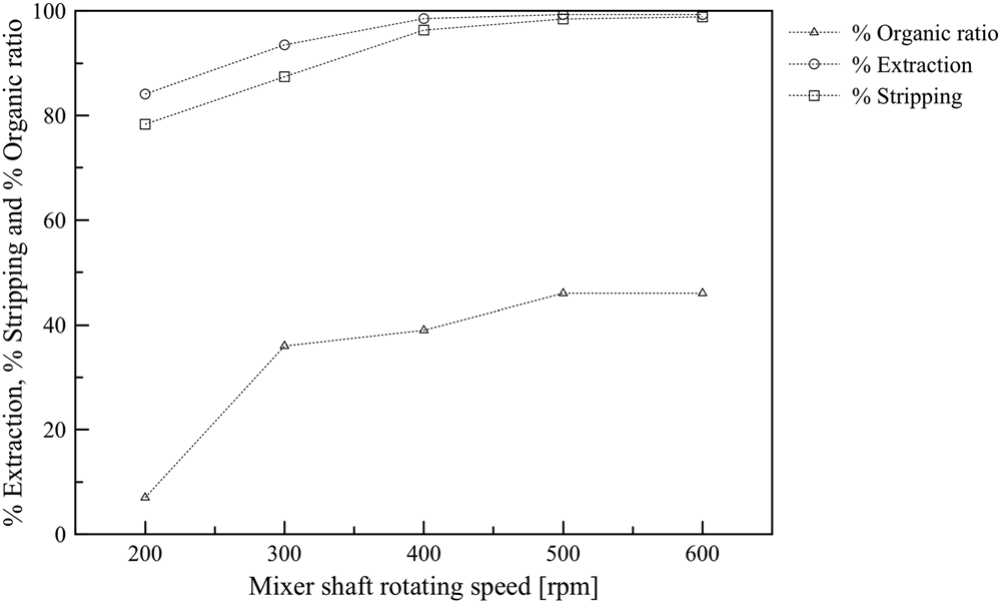
The effects of stirring speed on percent extraction and percent stripping, Y(III) concentration in feed phase = 1 mM feed agents = 0.5 M HNO_3_, stripping agents = 3.0 M HNO_3_, D2EHPA concentration in kerosene = 0.1 M, the flow rate of feed:stripping dispersion solution = 300:200 mL/min, stirring speed = 500 rpm and operating time = 60 min in circulating mode.

The influence of the stirring speed of the strip dispersion phase was studied to optimize uniform mixing of aqueous strip phase and to minimise thickness of the aqueous boundary layer. The stirring speed determines the degree of mixing between organic and aqueous phases and also influences dispersivity of aqueous strip solution in the strip dispersion phase. High dispersivity benefits separation performance^36,37^. Here, the effect of strip dispersion stirring speed on Y(III) extraction efficiency was examined. As shown in Fig. 5, as the stirring speed increased from 200 rpm to 400 rpm, percent extraction of Y(III) increased monotonically from 77 to 97%. Besides, the percent stripping showed a similar trend. It can be explained that the increase of stirring speed increased the dispersivity of aqueous strip solution in the strip dispersion phase and also increased the contact area between the two phases. However, the further increase in the stirring speed beyond 400 rpm had less effect on the percent extraction and stripping. The results indicated that at 400 rpm the minimum thickness of continuous phase boundary layer and the maximum of the overall mass transfer coefficient of the stirring speed had been reached.

**Figure 6:**
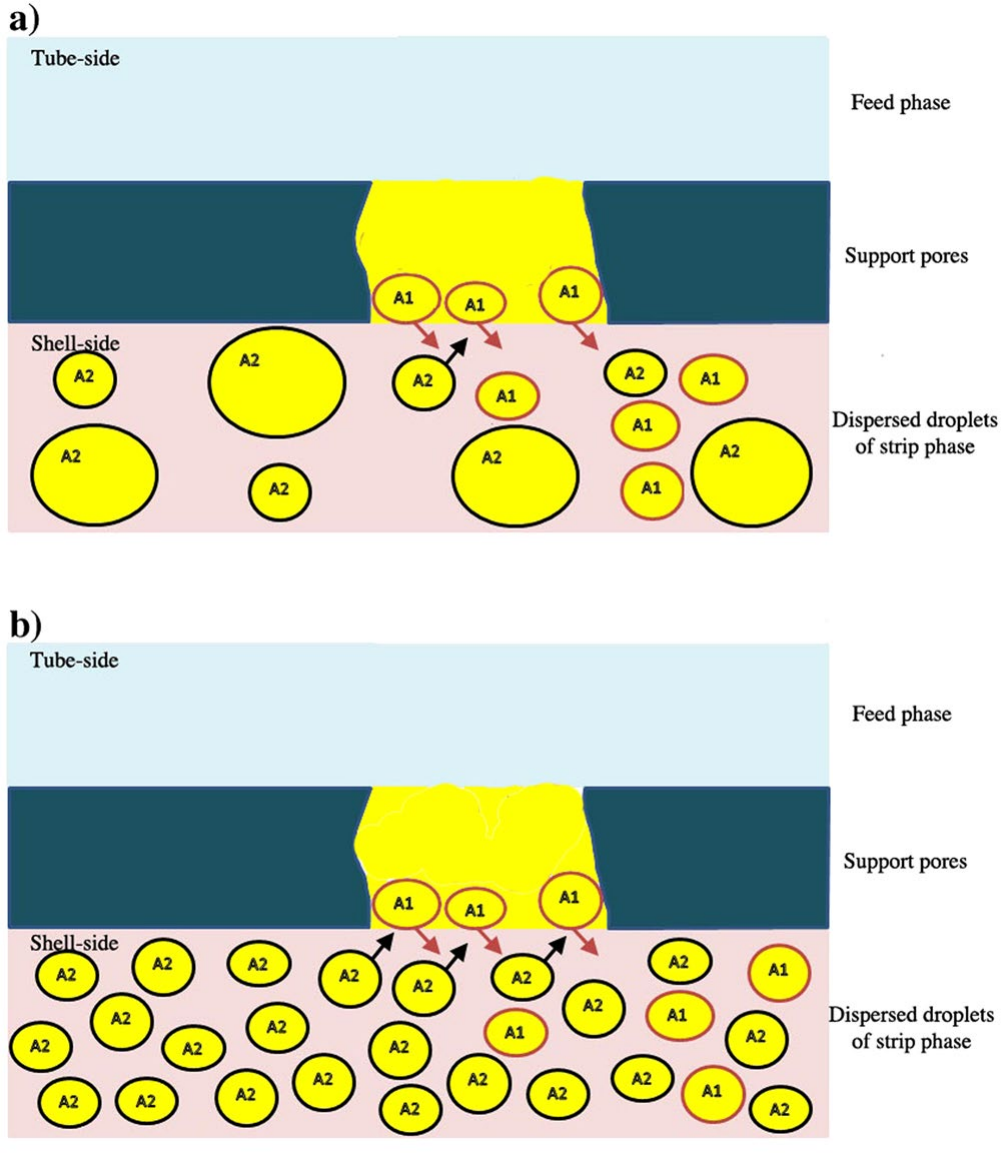
Schematic diagram for the strip dispersion phase; (**a**) stirring speed 200 rpm, (**b**) stirring speed 500 rpm.

Schematic diagram of the liquid membrane and the strip dispersion phase is shown in Fig. 6. The stirring speed affects size, dispersivity and the number of droplets of aqueous strip solution the strip dispersion phase. The increase of the stirring speed resulted in the increment of the interfacial area as well as the rate of renewal and regeneration of liquid membrane phase. The droplets of the aqueous strip solution dispersed in a continuous organic phase are a water-in-oil emulsion. Besides, these droplets continuously replenished the liquid membrane phase by constantly supplying the organic membrane solution to the micropores of the supported hollow fiber. The constant supply of the organic liquid ensures stable and continuous operation of the PEHFSD system and diffusing of Y(III) complex to the interface between the aqueous strip solution and the organic liquid from the micropores. In this way, the direct contact between the aqueous strip solution and the organic liquid provides efficient mass transfer for stripping.

### Effect of feed flow rate

**Figure 7:**
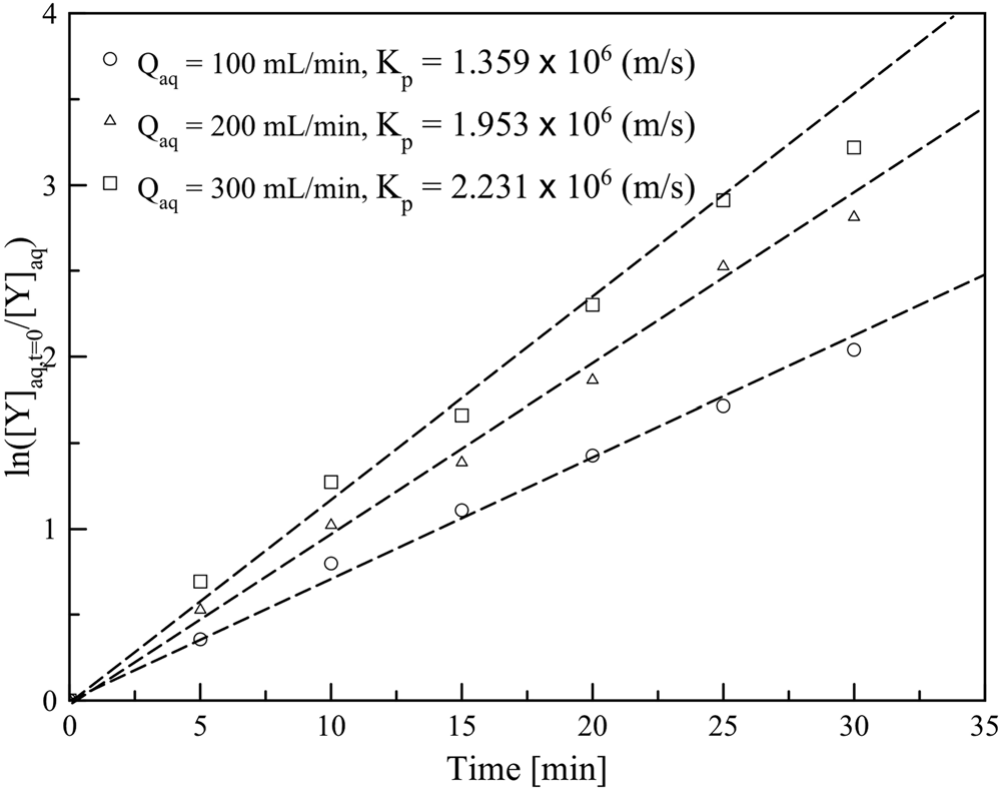
The effects of flow rate of feed phase versus time, Y(III) concentration in feed phase = 1 mM feed agents = 0.5 M HNO_3_, stripping agents = 3.0 M HNO_3_, D2EHPA concentration in kerosene = 0.1 M, the flow rate of feed stripping dispersion solution = 300:200 mL/min, stirring speed = 500 rpm and operating time = 30 min in circulating mode.

A series of experiments was performed to examine sufficient hydrodynamic conditions. The effect of feed flow rate on the transport of Y(III) is presented in Fig. 7. The increase in the aqueous flow rate from 100 to 200 mL/min, increased the transport of Y(III) and the further increase in the flow rate from 200 to 300 mL/min had less effect on the transport of Y(III). Besides, the overall volume mass transfer coefficient increased from 1.359 × 10^6^ to 1.953 × 10^6^ and to 2.231 × 10^6^ m/s. The increase of the transport of Y(III) has resulted from the increment of the interfacial area and the reduction of the continuous phase boundary layer thickness^38^. Also, the increase of feed flow rate can supply more Y(III). However, a further increase in the aqueous flow rate was found to have a slight effect on the kinetics of Y(III) transport.

### Stability of PEHFSD and HFSLM system

To investigate the stability of PEHFSD and HFSLM systems, the profiles of Y(III) concentration in the feed and strip dispersion phases were studied under the fixed operating condition in five experiments. In PEHFSD, the strip dispersion phase was pumped through the shell-side of the module, and the feed phase flowed through the tube-side. The flow rate of feed and dispersion phases were maintained at 300 mL/min. The concentration of HNO_3_ in the feed was 0.5 M. The initial concentration of Y(III) was 1 mM. The volumetric ratio of membrane solution/stripping solution in the feed phase was 1. In the dispersion phase, the HNO_3_ concentration was 3 M and the concentration of D2EHPA was 0.1 M. In HFSLM, the feed phase was pumped through the tube-side, and the strip phase was fed through the shell-side. The volume flow rate of feed and strip phases was maintained at 300 mL/min throughout the experiment. The concentration of HNO_3_ in the feed was 0.5 M. The initial concentration of Y(III) was 1 mM. The volumes of feed solution and stripping solution were 1 L. The HNO_3_ concentration in the strip phase was 3 M. The concentration of D2EHPA was 0.1 M.

**Figure 8:**
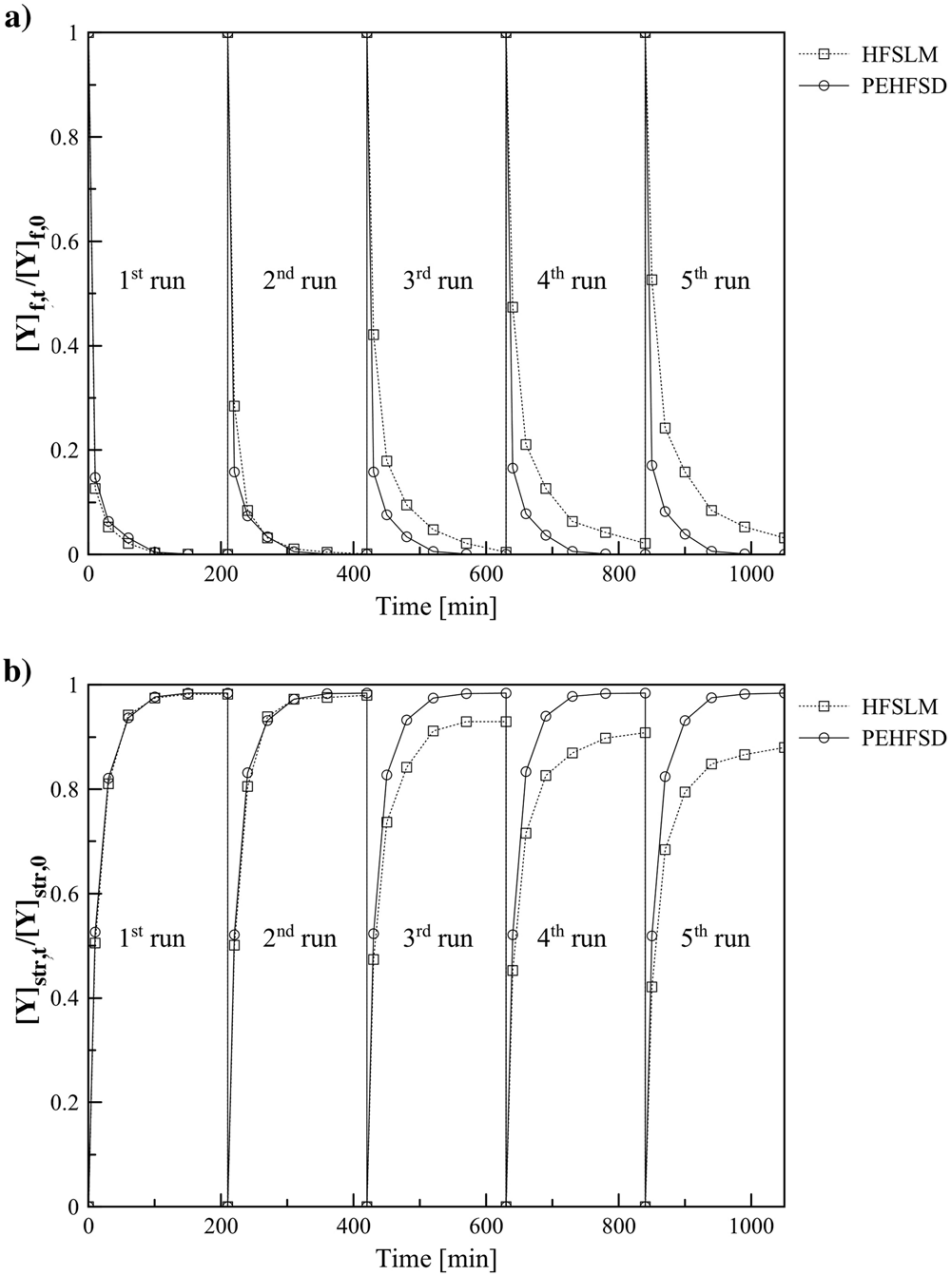
(**a**) The stability of PEHFSD and HFSLM system in feed phase, Y(III) concentration in feed phase = 1 mM feed agents = 0.5 M HNO_3_, stripping agents = 3.0 M HNO_3_, D2EHPA concentration in kerosene = 0.1 M, the flow rate of feed: stripping dispersion solution = 300:200 mL/min, stirring speed = 500 rpm and operating time per batch = 60 min in circulating mode, (**b**) The stability of PEHFSD and HFSLM system in the strip phase.

The results are shown in Fig. 8, apparently, the membrane solution in PEHFSD can be reused many times before the re-extraction with the carrier after each experiment. The membrane solution in HFSLM can also be reused, but the tendency of the concentration ratio of Y(III) in the feed solution was stable only in two experiments. After three experiments, the concentration ratio of Y(III) in the feed solution decreased gradually. On the contrary, the membrane module of PEHFSD system can be reused many times, and the tendency of the concentration ratio of Y(III) in the feed solution remained stable throughout all five experiments. As presented in Table 1, the *K*_*p*_ value of PEHFSD system was stable in five experiments, but the *K*_*p*_ value of HFSLM system was stable in two experiments. After five experiments of HFSLM system, the percent extraction of Y(III) decreased from 99.97 to 96.84 and percent stripping of Y(III) dropped from 98.42 to 88.02. On the other hand, percent extraction and percent stripping of Y(III) in PEHFSD system remained constant about 99.97 and 98.40, respectively. This is because the carrier in traditional HFSLM system gradually loses with time. However, in PEHFSD system, the dispersed droplets regularly supplied the organic membrane solution to the micropores of the supported hollow fiber. Thus, the PEHFSD system outperformed HFSLM system concerning stability.

**Table 1:** The stability HFSLM and PEHFSD system.

Run (cycle)	1	2	3	4	5
*K*_p_×10^6^ (m/s) of HFSLM system	8.505	6.728	5.126	3.800	3.569
*K*_p_×10^6^ (m/s) of PEHFSD system	8.400	8.357	8.320	8.227	8.184
Percent extraction of Y(III) in HFSLM system	99.97	99.89	99.57	97.89	96.84
Percent extraction of Y(III) in PEHFSD system	99.97	99.97	99.96	99.95	99.95
Percent stripping of Y(III) in HFSLM system	98.21	98.01	92.94	89.95	88.02
Percent stripping of Y(III) in PEHFSD system	98.42	98.42	98.41	98.40	98.38

### Comparison of HFSLM and PEHFSD system

**Figure 9:**
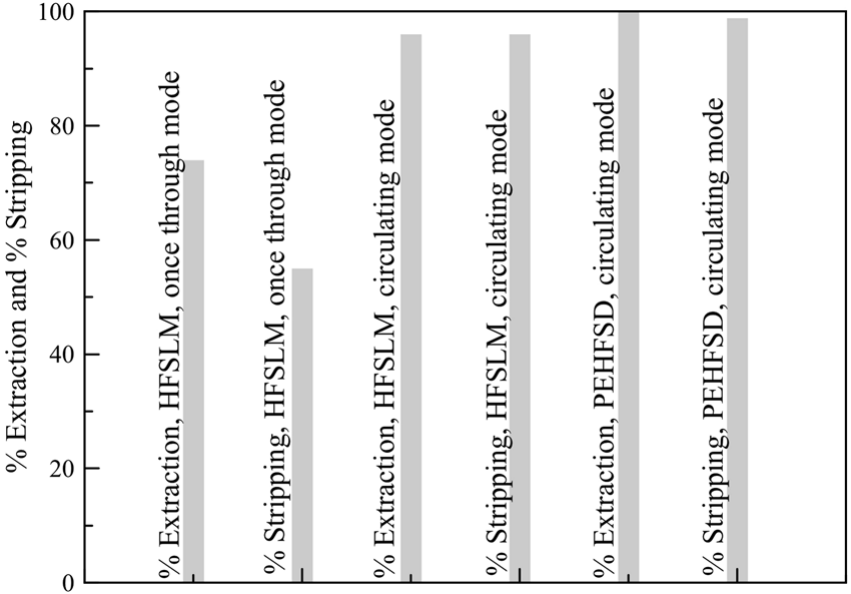
Comparison of HFSLM and PEHFSD system.

The comparison of liquid membrane system was performed under the optimal conditions (Table 2). The results are shown in Fig. 9, the PEHFSD system exhibited 99% extraction and 98% stripping. In comparison, the HFSLM system showed the percentages of extraction and stripping 96% and 95%, respectively. Also, Table 2 shows the mass transfer coefficient (*K*_p_) of HFSLM and PEHFSD systems in the circulating mode. The results indicated that the separation of PEHFSD system was better than HFSLM system. In the case of HFSLM system, the extraction and stripping occur at the respective liquid/liquid interfaces immobilized at their respective mouths of micropores in the polymeric support, and the liquid membrane phase is only in the micropores. The relatively small volume of the liquid membrane phase, interfacial shear force/emulsification and osmotic pressure difference are the major reasons for the instability of a supported liquid membrane^39,40^. In PEHFSD system, the liquid membrane layer is stabilized by a constant supply of the organic solution to the membrane micropores. This system developed the technique of SLM with strip dispersion (PEHFSD) to address the instability issue of the traditional HFSLM^41,42^. Thus, we can conclude that separation of PEHFSD system outperformed HFSLM system.

**Table 2:** The optimum condition of each system.

Parameter	HFSLM	HFSLM	PEHFSD
Mode	once through	circulating	circulating
Feed acidity	H_2_NO_3_ pH_5_	H_2_NO_3_ 0.05 M	H_2_NO_3_ 0.5 M
Feed concentration (g/L)	N/A	1	0.112
Feed flow rate (mL/min)	100	100	300
Carrier type	TBP and Cyanex 272	DNPPA	D2EHPA
Carrier concentration (M)	0.2:0.4	0.2	0.1
Organic solvent	Kerosene	Kerosene	Kerosene
Stripping acidity	H_2_NO_3_ 0.8 M	H_2_SO_4_	H_2_NO_3_
Striping flow rate (mL/min)	100	100	200
Reference	Ramakul *et al*.^43^	Vijayalakshmi *et al*.^44^	This study
*K*_p_ × 10^6^ (m/s)	N/A	1.921	8.402

## Conclusion

The PEHFSD system was found to be suitable for the simultaneous extraction and recovery of Y(III) from acidic nitrate medium in a single contactor using D2EHPA in kerosene as the carrier. The analysis of Y(III) transport was done on the concentration ratio of Y(III) ([Y]_*t*_/[Y]_0_), percent extraction, percent stripping and overall mass transfer coefficient (*Kp*). The effects of initial feed concentration, ratio of aqueous feed phase and strip dispersion phase, stirring speed, feed flow rate and type of diluent were examined to determine the optimal operating condition. Most importantly, the PEHFSD system exhibited higher performances than the HFSLM system concerning both stability and separation.

### Data Availability

The authors declare that the main data supporting the findings of this study are available within this article and its Supplementary Information.

## Electronic supplementary material

Supplementary Information
