# Supplementary material for: Yttrium (III) Recovery with D2EHPA in Pseudo-Emulsion Hollow Fiber Strip Dispersion System
Source: Sci Rep. 2018 May 16;8:7627. doi: 10.1038/s41598-018-25771-4 (PMC5955940; doi:10.1038/s41598-018-25771-4)
Supplement: Supplementary file 1 — Supplementary Information [file 41598_2018_25771_MOESM1_ESM.pdf]

Supplementary Information for Yttrium (III) Recovery with D2EHPA in Pseudo-Emulsion Hollow Fiber Strip Dispersion System

Teeprapon Pirom[1], Amornchai Arponwichanop[1], Ura Pancharoen[1], Tetsu Yonezawa[2], and Soorathep Kheawhom[1,*]

[1]Computational Process Engineering Research Laboratory, Department of Chemical Engineering, Faculty of Engineering, Chulalongkorn University, Bangkok 10330, Thailand

[2]Division of Materials Science and Engineering, Faculty of Engineering, Hokkaido University, Kita 13 Nishi 8, Sapporo, Hokkaido, 060-8628, Japan

[*]soorathep.k@chula.ac.th

Properties	Descriptions
Material	Polypropylene
Inside diameter of hollow fiber	240 μm
Outside diameter of hollow fiber	300 μm
Effective length of the hollow fiber	15 cm
Number of hollow fibers	35,000
Average pore size	0.03 μm
Porosity	25%
Membrane surface area	1.4×10^4 cm
Operating pressure	2.1-7.2 bar
Operating temperature	278-343 K

Table S1: Physical properties of the hollow fiber module.

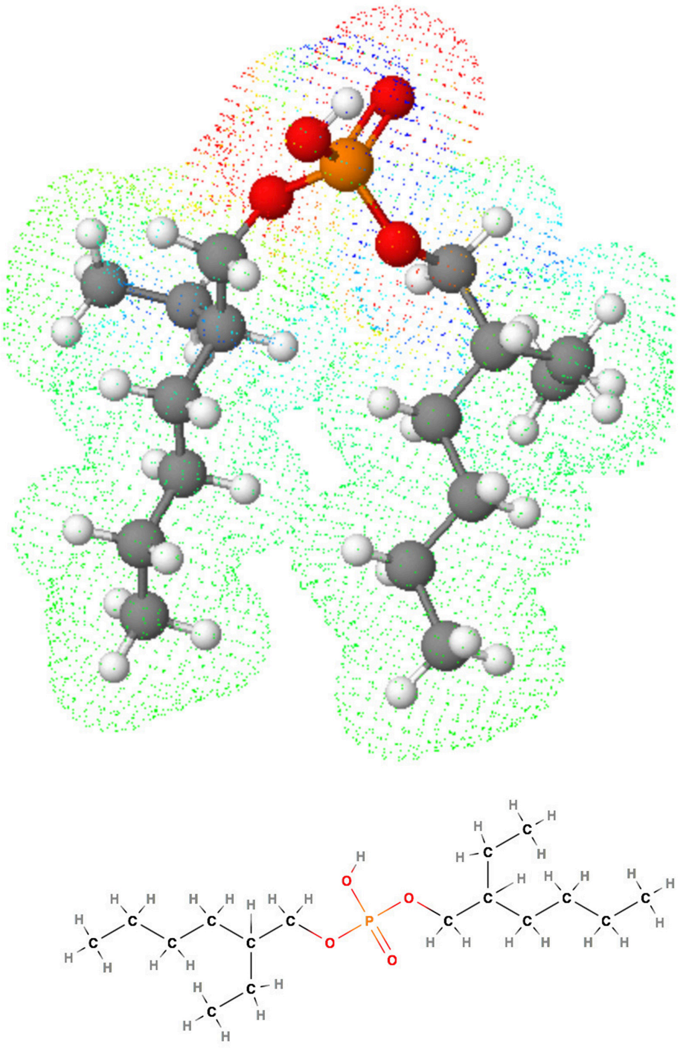

Figure S1: The molecular structure of Di(2-ethylhexyl)phosphoric acid (D2EHPA).

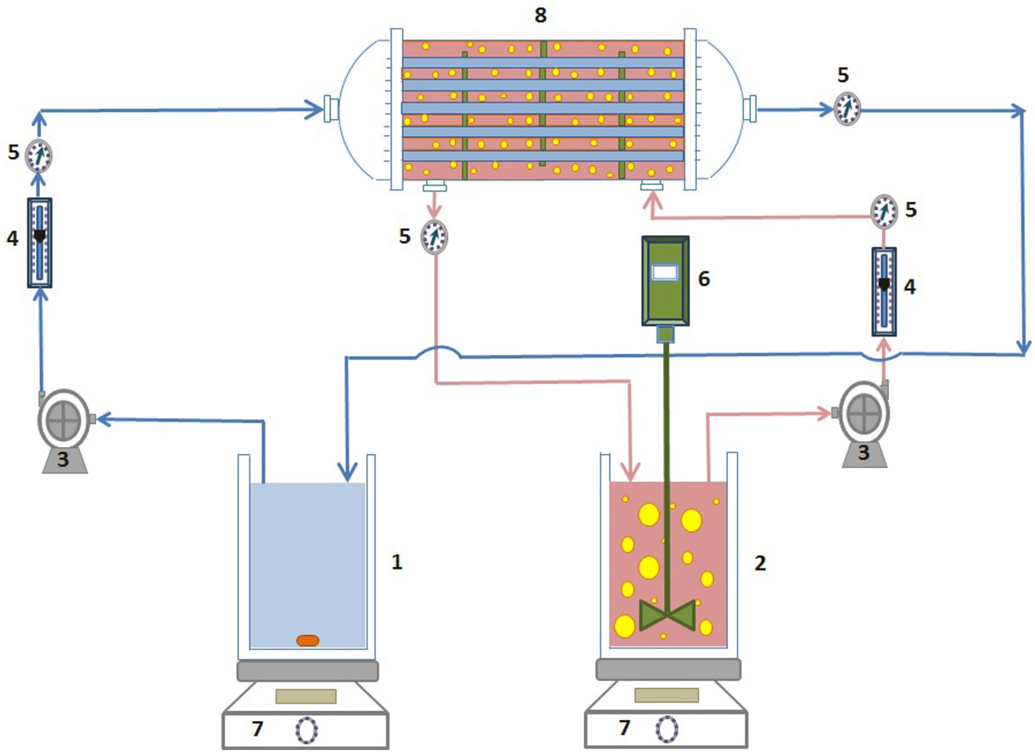

Figure S2: Schematic diagram of the PEHFSD system.

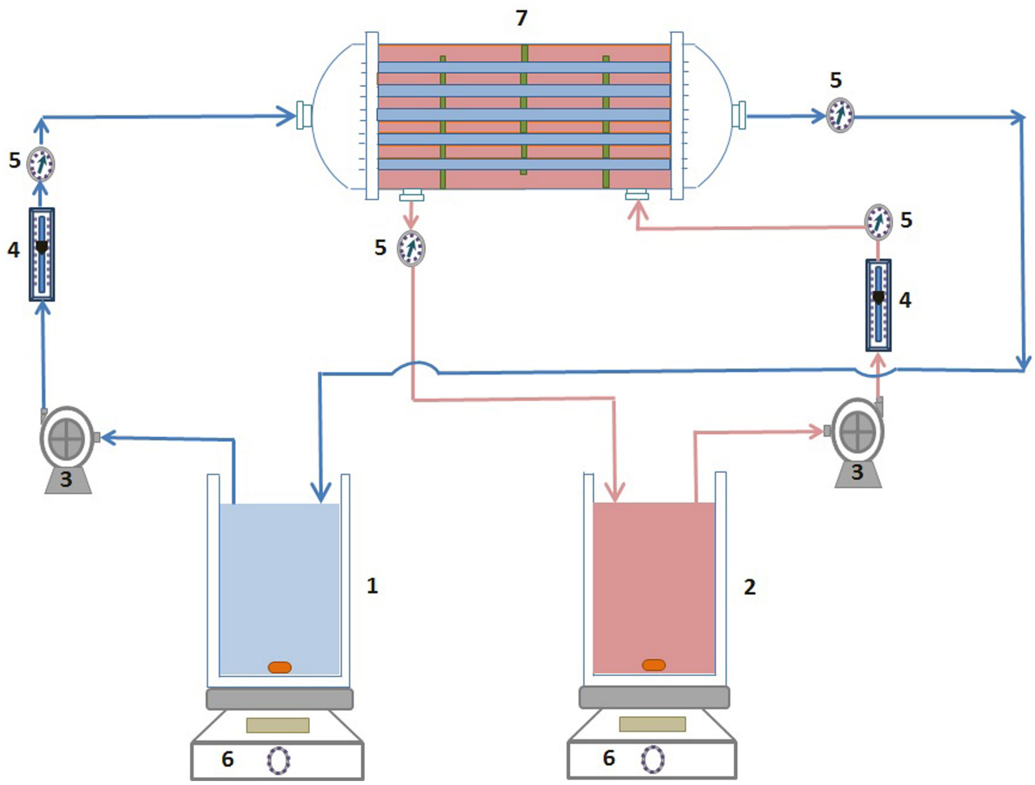

Figure S3: Schematic diagram of the HFSLM system.
